# Making research and evaluation more relevant and useful in the real world: favoured solutions and uncomfortable realities

**DOI:** 10.1186/1472-6963-14-S2-O1

**Published:** 2014-07-07

**Authors:** Nicholas Mays

**Affiliations:** 1 London School of Hygiene & Tropical Medicine, UK

## 

There has been a recent upsurge of advocacy from trialists and policy ‘modernisers’ for far more use of RCTs as the basis for health and wider public policy. This is exemplified by the UK Cabinet Office’s report *‘Test*, *Learn*, *Adapt’* (2012). Mainstream policy makers are now being told that they should make policy by experimenting like scientists. Drawing on experience as an applied health services researcher and policy adviser in government, I will attempt to stimulate reflection on the following questions: how can we explain the timing of this phenomenon; how realistic and *helpful* is it; and where does it leave the contribution of evaluation in policy?

